# MicroRNA Biogenesis is Enhanced by Liposome-Encapsulated Pin1 Inhibitor in Hepatocellular Carcinoma

**DOI:** 10.7150/thno.34588

**Published:** 2019-07-09

**Authors:** Dan Sun, Shuangyan Tan, Yanli Xiong, Wenchen Pu, Jiao Li, Wei Wei, Canhua Huang, Yu-Quan Wei, Yong Peng

**Affiliations:** 1 State Key Laboratory of Biotherapy and Cancer Center, National Clinical Research Center for Geriatrics, West China Hospital, Sichuan University, Chengdu, 610041, Sichuan, China.; 2 Key Laboratory of Bio-Resource and Eco-Environment of Ministry of Education, College of Life Sciences, Sichuan University, Chengdu 610065, China.; 3 West China School of Basic Medical Sciences & Forensic Medicine, Sichuan University, Chengdu 610041, China.

**Keywords:** hepatocellular carcinoma, Pin1, API-1, liposome, targeted therapy

## Abstract

Hepatocellular carcinoma (HCC) is in an urgent need of new, effective therapies to reduce morbidity and mortality. We have previously demonstrated that peptidyl-prolyl cis/trans isomerase Pin1 is a potential target for HCC therapy, due to its pivotal role in HCC development through regulating miRNA biogenesis, and discovered the small molecule API-1 as a novel and specific Pin1 inhibitor. Despite its significant anti-HCC activity, the low water solubility and *in vivo* bioavailability of API-1 limit its clinical application. To address these issues, we herein developed a liposomal formulation of API-1 to improve API-1 delivery and enhance its anti-HCC efficacy.

**Methods**: We designed and developed a nanoscale liposomal formulation of API-1, named as API-LP. Subsequently, the mean diameter, polydispersity, zeta potential, encapsulation efficiency and thermal properties of the optimization API-LP were characterized. The enhanced anti-HCC activity and the molecular mechanism of API-LP were investigated both *in vitro* and *in vivo*. Finally, the safety and pharmacokinetic property of API-LP were evaluated systematically.

**Results**: API-LP had good formulation characteristics and exhibited an enhanced *in vitro* activity of suppressing proliferation and migration of HCC cells when compared with free API-1. The mechanism study showed that API-LP upregulated miRNA biogenesis via inhibiting Pin1 activity followed by restoring the nucleus-to-cytoplasm export of XPO5. Because of the increased delivery efficiency, API-LP displayed a stronger ability to promote miRNA biogenesis than free API-1. Importantly, API-LP displayed higher systemic exposure than free API-1 in mice without apparent toxicity, resulting in an enhanced tumor inhibition in xenograft mice.

**Conclusion**: The development and assessment of API-LP provide an attractive and safe anti-HCC agent, highlighting the miRNA-based treatment for human cancers.

## Introduction

Hepatocellular carcinoma (HCC) is the third leading cause of cancer death and the fifth most prevalent malignancy worldwide 1. Despite great advances in current treatments against HCC 2, relapse and metastasis are frequently observed in patients, and the 5-year survival rate remains quite low 3,4. Thus, it is in an urgent need to develop new effective therapies to decrease the morbidity and mortality.

MicroRNA (miRNA) is a class of small non- coding RNAs with 21 to 23 nucleotides in length and regulates gene expression at the post-transcriptional levels through repressing protein translation or destabilizing target mRNAs 5. In HCC, global miRNA expression is downregulated, which may be caused by comprised miRNA biogenesis 6. Under normal physiological conditions, miRNA biogenesis is tightly controlled at multiple steps 7-10, among which the nucleus-to-cytoplasm export of precursor miRNA (pre-miRNA) by exportin-5 (XPO5) is a rate-limiting step 11-14. Recently, we disclosed that XPO5 phosphorylation by ERK kinase and subsequent isomerization by prolyl isomerase Pin1 impair miRNA biogenesis, thus decreasing miRNA expression and promoting HCC development 15. Increasing evidences demonstrate that Pin1 is widely overexpressed in human cancers including HCC and participates in multiple cancer-associated signaling pathways 16-19. Therefore, Pin1 is an attractive target for HCC treatment.

Several early stage-developed Pin1 inhibitors, such as juglone, epigallocatechin gallate (EGCG), PiB and phenyl imidazole, have limited specificity and potency 20. Recently, all trans retinoic acid (ATRA) and the juglone analogue KPT-6566 were discovered as Pin1 inhibitors and exerted good anti-tumor effects 21-22. However, both molecules cause risky side- effect and toxicity, restricting their further development 23. API-1, a 6‑O-benzylguanine derivative, is a potent and specific Pin1 inhibitor identified by our team via the computer-aided virtual screening and *in vitro* PPIase activity assay, effectively suppressing cell proliferation *in vitro* and *in vivo*, at least in part, by modulating miRNA biogenesis 15.

Although API-1 is a potential drug candidate for HCC treatment, especially for Pin1-overexpressing and/or ERK-activated HCC, its clinical application is hampered by its low aqueous solubility and instability under acidic condition, leading to the poor *in vivo* bioavailability and attenuated therapeutic effects. Nanocarriers, such as liposomes, polymer nanoparticles and biodegradable microspheres, are potential approaches to improve the poor biopharmaceutical properties and drug delivery 24-27. Among them, liposomal formulation, with promising biosafety, is able to improve the bioavailability and effect of drugs toward certain tumors 28,29, prompting us to develop the liposome-encapsulated API-1 (API-LP) and investigate its pharmacokinetics, pharmacodynamics, toxicity, and anti-HCC efficacy.

## Experimental Section

### Materials

DMPC, DMPG, and DSPE-PEG_2000_ were purchased from the Lipoid Company, Germany; CHOL and Sephadex G-25 were obtained from Sigma; anti-DNMT1 antibody was purchased from New England Biolabs; anti-XPO5, anti-ERK, anti-p-ERK, anti-STAT3, anti-c-Myc and anti-GAPDH antibodies were purchased from Cell Signaling Technology. Antibody to the Ser416 phosphorylation site of XPO5 (anti-p-XPO5) was generated by Lifetein LLC and applied in our previous study 11.

All other reagents were of analytical reagent grade; water referred to ultrapurified Milli-Q water (Millipore, France) with a resistivity of ≥18 MΩ•cm.

### Cell lines and animals

SK-Hep1 cells were cultured in DMEM medium supplemented with 10% fetal bovine serum. Female BALB/c nude mice were purchased from Beijing Huafukang Bioscience (China). All animal experiments were approved by the Experimental Animal Ethics Committee of West China Hospital of Sichuan University (Chengdu, China).

### Preparation and characterization of liposomal formulation

Liposomal formulation of API-1 (API-LP) was prepared by thin-film evaporation 30,31. Single factor method was adopted for optimizing liposomal formulation. The optimal prescription of lipids was DMPC: DMPG: CHOL: DSPE-PEG_2000_ (22.5: 22.5: 50: 5 molar ratio). In details of API-LP preparation, API-1 and lipids were dissolved in chloroform and methanol mixture (1:1, v/v) with a drug/lipids ratio of 30% (W/W). The mixture was evaporated at 40 °C and 125 rpm in a rotary evaporator for 1 h to form a dry lipid film, hydrated for 1.5 h with 1 × PBS (pH 7.4) at 42 °C and 80 rpm, followed by ultrasonication in an ice-water bath for 10 min to evenly shrink the particle size. The final lipid concentration was 10 mg/ml. The unentrapped API-1 was removed by Sephadex G-25. Empty liposomal formulation (LP) was prepared using the same protocol but excluding API-1.

The encapsulation efficiency (EE) and loading efficiency of API-1 in API-LP were calculated according to the following equations:

Entrapment efficiency (%) = C_e_/C_t_ × 100%

Where C_t_ stands for the initial drug amount added, C_e_ stands for the drug amount encapsulated in the liposomes.

Loading efficiency (%) = (weight of encapsulated API-1/weight of lipid amount in the liposomes) × 100%

The average size distribution and zeta potential of liposomes were measured by a laser particle size analyzer (Malvern Nano-ZS 90, UK) utilizing dynamic light scattering (DLS) technique. Samples were diluted using water and measured in triplicate.

The morphology was observed by high resolution transmission electron microscope (TEM). The samples were prepared by placing the diluted liposomes on a copper grid followed by drying under reduced pressure and staining with 3% phosphotungstic acid for 30 seconds.

For stability assessment, API-LP was stored at 4 °C for four weeks. At different time points over the storage (i.e., Days 0, 1, 3, 5, 7, 14, and 28), the changes of particle size, PDI and entrapment efficiency of API-LP were measured.

### *In vitro* drug release

*In vitro* release of API-1 from API-LP was investigated in a simulated blood pH environment (pH 7.4). Briefly, 1 mL API-LP diluted ten times in advance was used to determine the initial API-1 concentration, and the remaining 9 mL was dialyzed in a dialysis bag (3500 Da) in 50 mL PBS (pH 7.4) containing 0.5% Tween-80. The experiment was performed in triplicate at 37°C with shaking at 100 rpm for one week. Samples were withdrawn at prescheduled sampling time points and replaced with the same volume of release buffer each time. The samples were quantified by high performance liquid chromatography (HPLC).

### Differential scanning calorimetry (DSC) spectra assay

DSC measurements were performed using NETZSCH DSC instrument. For DSC measurement, 5 to 8 mg of all powder or lyophilized liposome samples, including API-1, LP, API-LP and the mechanical mixtures of API-1 and LP (LP + API-1), were weighed respectively into the aluminum pans and sealed. Samples were scanned from -20 °C to 320 °C with 10 °C/min incremental increases.

### Cell viability

MTT assay was conducted to estimate cell viability. Briefly, SK-Hep1 cells were seeded in a 96-well culture plate (2000 cells/well) and then treated with different concentrations of API-LP or free API-1 at 72 h in a 5% CO_2_ incubator for indicated time. At the end of incubation, 10 µL of MTT solution (5 mg/mL) was added into each well. After a 3 h incubation, DMSO (100 μL) was added to dissolve the resultant purple formazan crystals. The absorbance was measured at 570 nm on Thermo Scientific Varioskan Flash Multimode Reader.

### Wound healing assay

SK-Hep1 cells were cultured in 6-well plates and wounded using a sterilized pipet tip to make a straight scratch. After being rinsed with 1 × PBS gently, cells were incubated in the medium containing DMSO, API-1 or API-LP (1 μM). Pictures were taken by an Olympus digital camera after 24 h and 48 h.

### Transwell migration assay

Cell migration ability was performed using Transwell insert chamber (Corning) of 24-well. SK-Hep1 cells were suspended in serum-free DMEM medium containing 5% BSA and seeded in the top chambers, whereas DMEM supplemented with 10% FBS was added into the bottom chambers. After 24 h, the cells in top chambers were fixed by 4% PFA for 20 min and stained with 1% crystal violet for 15 min. Graphic images were recorded using microscope.

### Cell cycle and apoptosis analysis by flow cytometry

Cell cycle and apoptosis were detected by flow cytometry assays. SK-Hep1 cells (2 × 10^5^ cells/well) were plated in a 6-well plate and treated with DMSO, free API-1 or API-LP (1 μM) for 72 h. For cell cycle analysis, cells were harvested with trypsin without EDTA to generate single cell suspensions and then washed once with ice-cold 1 × PBS. After that, cells were fixed overnight in 70% ice-cold ethanol. The fixed cells were washed once with ice-cold 1 × PBS, stained with PI solution (50 mg/mL PI, 0.1% NP-40, 0.1% sodium citrate, 0.1% Triton X-100), and then analyzed on a FACS Calibur (BD Biosciences).

APC-Annexin V with 7-AAD Apoptosis detection Kit (BioLegend) was used for direct observation of viable, early apoptotic, and late apoptotic cells. Cells were harvested and washed with ice-cold 1 × PBS, then stained with APC-Annexin V and 7-AAD following the manufacturer's instructions for apoptosis assay. The apoptosis ratio was analyzed using a FACS Calibur (BD Biosciences).

### Hemolysis assay

Healthy rabbit erythrocytes were collected, washed, and resuspended in normal saline to a 2% final concentration. The LP and API-LP at final concentration of 1.25, 2.5, 3.75, 5 and 6.25 mg/ml were added to the erythrocyte suspension, respectively. And incubated at 37 °C with shaking at 80 rpm for 3 h followed by centrifugation at 1500 rpm for 10 min. Hemoglobin release in the supernatant was measured by UV spectrophotometry at 545 nm to calculate the percentage of hemolyzed cells. Less than 5% hemolysis stands for a good hemocompatibility and non-toxicity against erythrocytes membranes.

### Safety study of the liposomes *in vivo*

Safety of the liposomes *in vivo* was evaluated by blood biochemical analysis, H&E staining and Masson staining of tissues. Briefly, following *i.v.* administration of normal saline, LP, API-1 or API-LP in the BALB/c nude mice, the peripheral blood samples were collected using heparin and centrifuged for serum biochemical analysis. The tissue samples were simultaneously excised and fixed in 4% PFA. Blood ALT, AST, TBIL, ALB, UREA and total protein analyses were performed using BioAssay Systems blood chemistry assay kits following the manufacturer's instructions. The tissue samples were embedded in paraffin, then cut into 5 μm thick paraffin sections and subsequently placed on glass slide. The slides were stained with H&E. The histopathological alterations were observed and imaged with a light microscope (OLYMPUS BX43).

In order to facilitate the judgment of the positivity and degree of hepatic injury of mice, an ace hepatic injury mice model was built by treating mice with tetracycline by gavage at dose of 500 mg/kg. Mice were fasted after oral tetracycline for 30 h and then were executed. The serum ALT, AST, TBIL and hepatic histopathological examinations were performed to evaluate liver injuries.

### Pharmacokinetic study of API-LP in mice

The pharmacokinetic performance of API-LP was assessed in BALB/c mice. Following overnight fasting, six mice were randomized into two groups and received *i.v.* injection of free API-1 in 1 × PBS (pH 7.4) with 5% DMSO and API-LP in 1 × PBS (pH 7.4), respectively, at 10 mg/kg dose. At the prescheduled sampling time points, 20 μL blood samples were collected through caudal vein bleeding into heparinized centrifuge tube, followed by the centrifugation to obtain plasma, which was then stored at -80 °C until determination of the drug concentration.

For API-1 quantitation, plasma samples were thawed to room temperature. API-1 was extracted using acetonitrile and quantified by HPLC with QTRAP 5500 tandem mass spectrometry (LC/MS/ MS) system 32. The pharmacokinetic parameters based on the measured concentrations were calculated using GraphPad Prism (GraphPad Software).

### Xenograft in nude mice and treatment

SK-Hep1 cells during the exponential-growth phase were collected and suspended in saline solution containing 50% Matrigel (Corning) at 5 × 10^7^/mL. Then 100 μL of cells were injected into female BALB/c nude mice (5 weeks old) subcutaneously. Tumor growth was recorded every 3 days, and the tumor volume was calculated as ab^2^/2 (a, long diameter; b, short diameter) 33. All mice were randomized into 4 groups (8 mice per group) when the tumor volume reached 150-200 mm^3^ and then were intravenously injected with normal saline, LP, free API-1 or API-LP at the 4 mg/kg dosage once every two days. At the end of the treatment, tumors were removed from mice and RNAs were extracted for miRNA detection.

### Immunofluorescence analysis

SK-Hep1 cells cultured on glass coverslips after different treatments were fixed in 4% PFA for 20 min, permeabilized with 0.5% Triton X-100 for 10 min, blocked with 5% BSA, and then incubated with primary anti-XPO5 antibody at 4 °C overnight, followed by incubation with Alexa Fluor dye- conjugated secondary antibodies for 1 h. Then the nuclei were stained with DAPI for 5 min at room temperature before mounting in Immu-Mout (Thermo fisher). Confocal fluorescence images were captured using Leica TCS SP5 II confocal spectral microscope.

### miRNA quantification by real-time PCR assay

Quantitative RT-PCR was used to assess miRNA expression levels. Total RNAs were extracted by TRIzol reagent (Invitrogen) from cells and transcribed by M-MLV reverse transcriptase (Invitrogen). Hairpin-it^TM^ real-time PCR miRNA kit for miR-122, let-7a, miR-29b and miR-146a were purchased from Shanghai GenePharma Co., Ltd (China). The miRNA levels were determined by Applied Biosystems StepOne Plus Real-Time PCR Systems using SYBR green master mix (Thermo fisher). Small endogenous nucleolar U6 RNA was used as internal control for miRNA normalization.

### Protein preparation and immunoblotting

For total protein preparation, cells were lysed on ice for 20 min in the RIPA lysis buffer (Beyotime) with protease inhibitor cocktail (Bimake), followed by centrifugation at 12000 rpm for 10 min at 4 °C. The resultant proteins in the supernatants were stored at -80 °C or directly subjected to SDS-PAGE. For nuclear-cytoplasmic fractionation of proteins, RLN buffer (5 mM Tris-HCl pH 7.4, 1.5 mM MgCl_2_, 140 mM NaCl, 0.5% NP-40) was freshly prepared when used. SK-Hep1 cells were harvested and gently resuspended in 200 µL RLN buffer with protease inhibitor cocktail, followed by centrifugation at 300 g for 2 minutes at 4 °C, the resultant supernatants are the cytoplasmic proteins. Then the pellets were gently washed twice with 400 µL RLN buffer and resuspended in 200 µL RLN buffer to get the nuclear proteins. Proteins were resolved on SDS-PAGE gels, transferred onto PVDF membranes, and then incubated with appropriate antibodies. The signals were detected using ECL reagents (Life Technologies).

### Immunohistochemistry

The experimental tissues were fixed with formalin and embedded in paraffin. Sections with 5 μm in thickness were prepared for H&E staining, Masson staining and immunostaining with anti-RIP3 antibody (Abcam). The images were acquired on an Olympus digital camera attached to a light microscope.

### Statistical methods

The data are presented as the mean ± SD of at least three independent experiments. One-way ANOVA or unpaired two tailed Student's t-test was used to establish statistical significance using GraphPad Prism (GraphPad Software). The *p*-value < 0.05 was considered statistically significant.

## Results and Discussion

### Optimization and characterization of API-1 liposomal formulation (API-LP)

Due to their advantages in efficient encapsulation of small molecule drugs with poor water solubility, the lipid components DMPC, DMPG and DSPE-PEG_2000_ were chosen to prepare liposome 34-37. Moreover, CHOL was used to enhance lipid component fluidity and further improve encapsulation efficiency 38-39. All liposomal formulations with different CHOL:lipids ratios and API-1:lipids ratios were prepared by the thin-film evaporation method 30,31. Based on our previous experience and current experiments, the DMPC:DMPG ratio for liposome preparation was optimized to be 1:1 (data not shown). The CHOL:lipids ratio of 50% was found to make API-1 liposome smaller diameter, lower polydispersity (PDI) and higher API-1 encapsulation efficiency (EE) than either 45% or 55% CHOL:lipids ratio (Table 1), and this ratio was fixed to further optimize API-1:lipids ratio. As shown in Table 1, the mean diameter of liposome increased from 86.6 to 268.1 nm with the increasing of API-1:lipids ratio from 0 to 40%; but the prescription with API-1:lipids ratio of 30% eventuated the optimal EE (65.21%) with the loading efficiency of 19.56%. Therefore, comprehensively considering the values of diameter, PDI and EE, the optimized API-1 liposomal formulation, named as API-LP, was confirmed as DMPC/DMPG/CHOL/ DSPE-PEG2000 (22.5:22.5:50:5) with API-1/lipids ratio of 30% (w/w).

**Figure 1:**
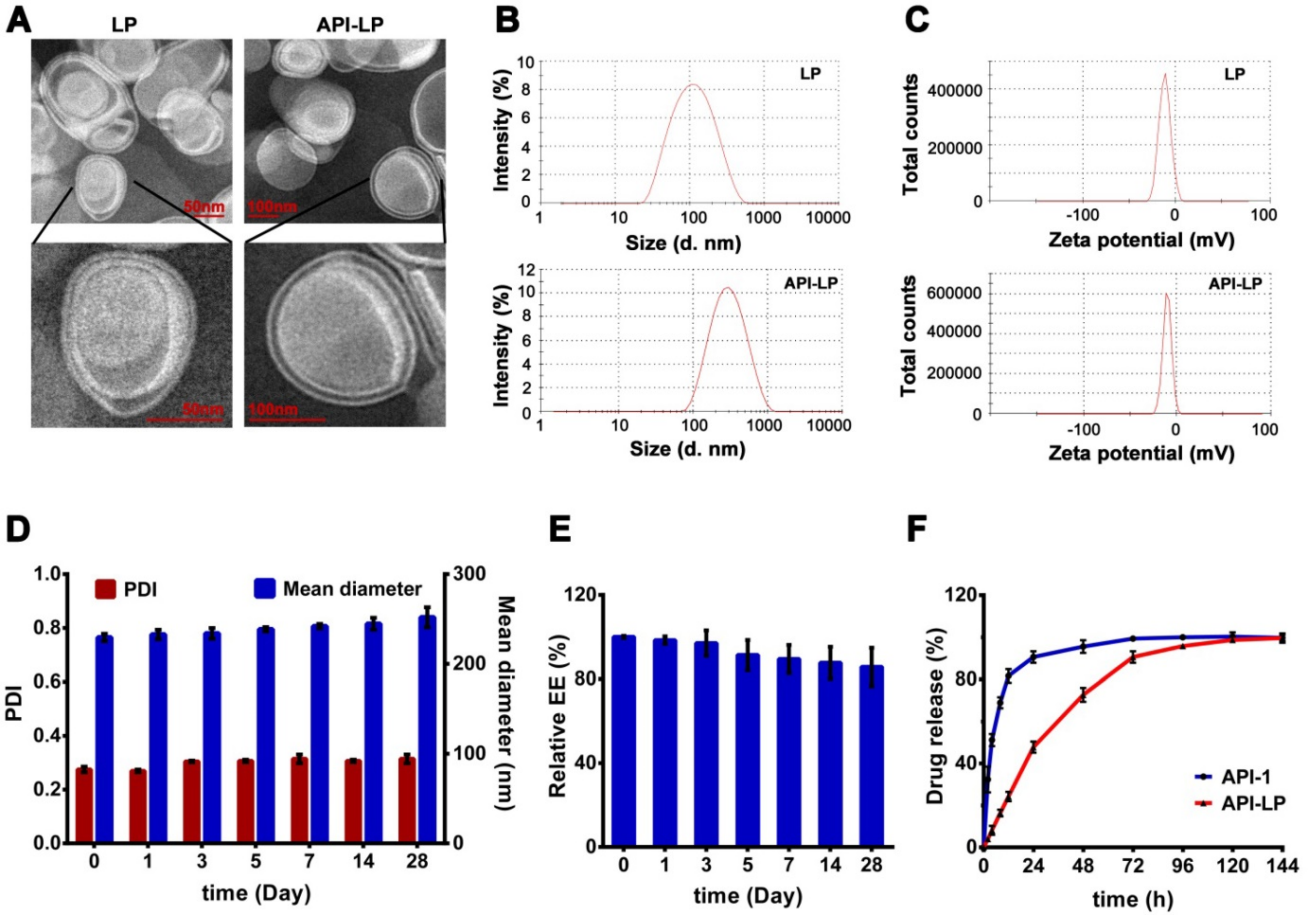
** Characterization of liposomal formulations.** (A) Representative TEM photograph of LP (left) and API-LP (right). (B-C) Size distribution (B) and Zeta potential (C) of LP and API-LP in DLS analysis. (D) Change of particle size and PDI of API-LP over storage at 4 °C. (E) Change of entrapment efficiency of API-LP over storage time at 4 °C. (F) Drug release from the free API-1 and API-LP.

The TEM imaging revealed that API-LP and the empty liposome LP had a typical finger-like liposome morphological structure and were spherical in aqueous solution (Figure 1A). The diameters measured by TEM and DLS were consistent, with 241.5 nm for the optimized API-LP formulation and 86.6 nm for LP, respectively (Table 1 and Figure 1B). All formulations had low PDIs (< 0.309) and were mildly negatively charged with zeta potential values of -10 to -13.4 mV (Table 1 and Figure 1C). When stored at 4 °C for over 4 weeks, the optimized API-LP formulation exhibited good stability with regard to diameter and PDI (Figure 1D) and the retained API-1 (Figure 1E). The loaded API-1 leaked only 15.7% over storage, which mainly occurred in the last 2 weeks (Figure 1E). Thus, API-LP was prepared and stored at 4 °C, and subjected to *in vitro* and *in vivo* experiments within 3 weeks. The *in vitro* release of API-LP was investigated by dialysis in a release medium (0.5% Tween-80 solution in PBS). As shown in Figure 1F, API-LP exhibited a slower release rate (24.2%) than free API-1 (81.6%) in the first 12 h of dialysis, suggesting a longer blood circulation.

**Figure 2:**
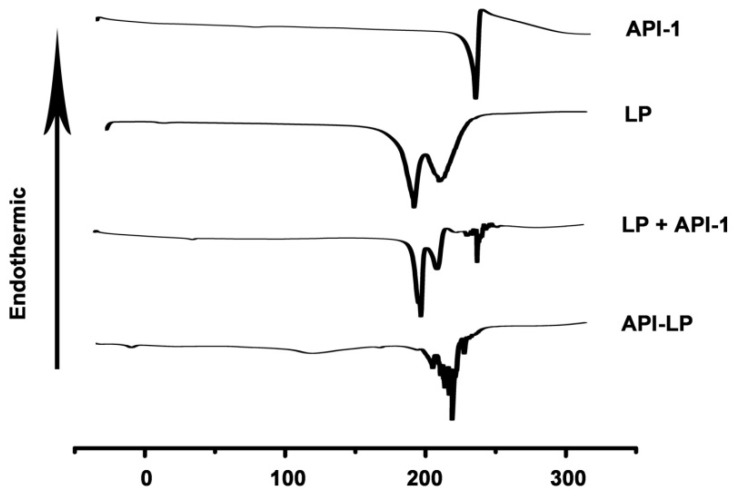
** The DSC thermograms.** DSC curves of the free API-1 (API-1), empty liposome (LP), the mixtures of LP and API-1 (LP + API-1), and API-1 liposomal formulation (API-LP) are presented.

The thermal properties of different samples were measured by DSC. As shown in Figure 2, API-1 showed a characteristic endothermic peak at 221.11 °C and an exothermic peak at 224.89 °C, whereas LP exhibited characteristic endothermic peaks at 183.43 °C and 210.16 °C. When LP and API-1 were mechanically mixed (LP + API-1), the DSC curve had major characteristic endothermic peaks for both. However, API-LP displayed a new endothermic peak at 226.64 °C, without the characteristic peaks of both API-1 and LP. These results indicated the interaction between API-1 and lipids in API-LP and demonstrated the success of API-1 loading to liposome. With confirmed physicochemical characteristics, the optimized API-LP formulation was adopted in the subsequent safety and efficacy evaluations.

### Enhanced anti-HCC activity of API-LP *in vitro*

**Figure 3:**
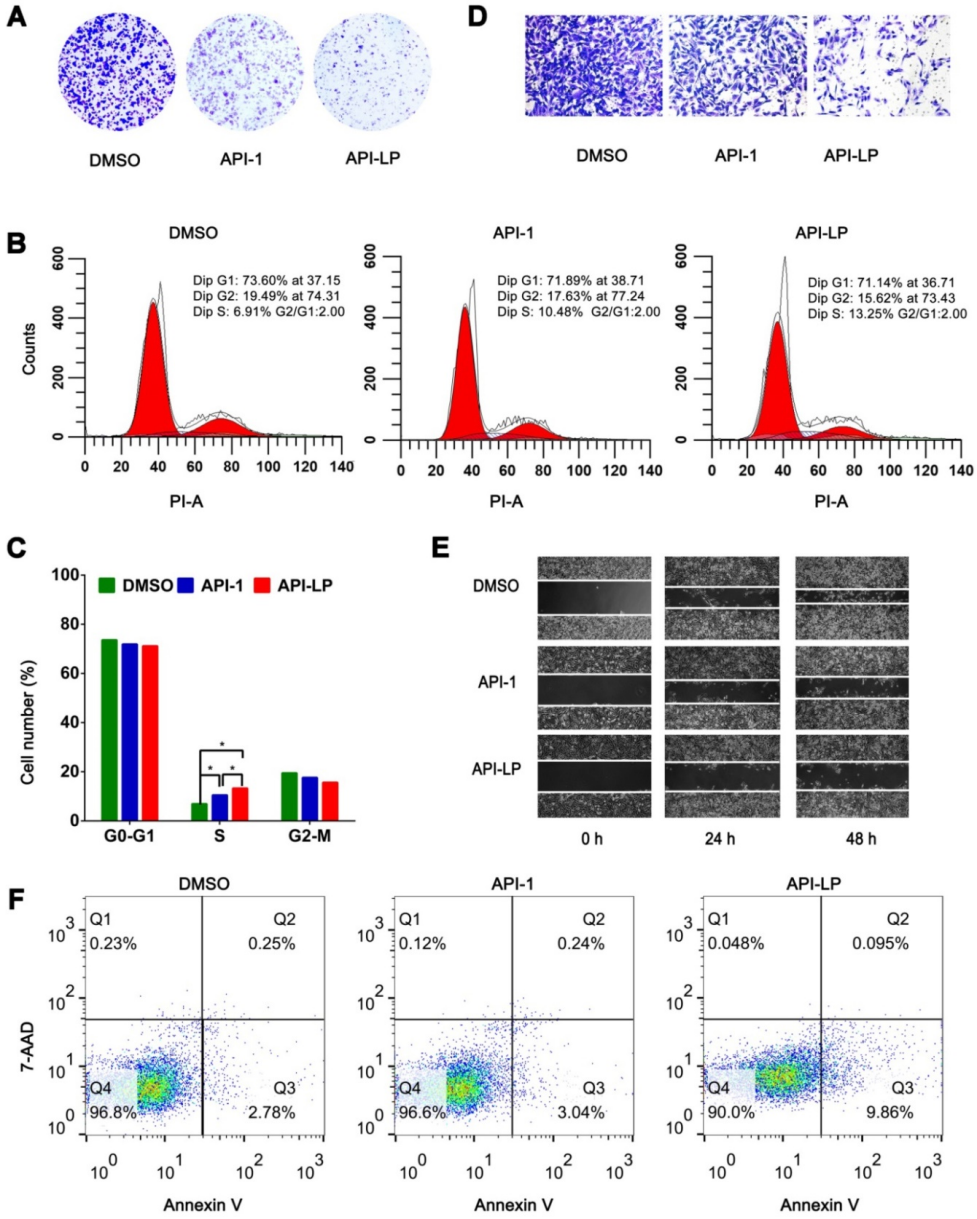
***In vitro* anti-HCC activity of API-LP.** (A) Colony formation assays in SK-Hep1 cells treated with DMSO, API-1 (1 μM) and API-LP (1 μM) for one week. (B-C) Cell cycle analysis by flow cytometry (B) and quantification (C) of SK-Hep1 cells incubated with DMSO, API-1 (1 μM) and API-LP (1 μM). Data were shown as the means ± SD, n=3. *p < 0.05. (D-F) Transwell migration assays (D), wound healing assays (E) and apoptosis analyses by flow cytometry (F) of SK-Hep1 cells treated with DMSO, API-1 (1 μM) and API-LP (1 μM).

To investigate whether API-LP has the advantages over free API-1 to inhibit HCC cell growth, MTT assays were performed. It was observed that both API-LP and free API-1 inhibited SK-Hep1 cell proliferation in a dose-dependent manner, while API-LP exhibited stronger inhibitory activity than free API-1 (Figure S1). This conclusion was further supported by colony formation experiments (Figure 3A). Flow cytometry indicated that the reduced proliferation upon API-LP treatment was caused by cell cycle arrest (Figure 3B and 3C), consistent with our previous results for free API-1 15. The data of Transwell migration experiment and wound healing assay indicated that API-LP had a stronger suppression of cell mobility in SK-Hep1 cells than free API-1 (Figure 3D and 3E). Intriguingly, API-LP induced early apoptosis in approximately 10% SK-Hep1 cells after 72 h incubation (Figure 3F). Taken together, the anti-HCC potency of API-1 (i.e., suppressing cell proliferation and migration) was significantly enhanced by liposomal formulation.

### API-LP increases the nucleus-to-cytoplasm export of pre-miRNAs with XPO5 to suppress HCC cell proliferation and metastasis

We recently reported that XPO5 phosphorylation by ERK kinase and the following isomerization by Pin1 isomerase in HCC cells lead to the retaining of XPO5 in the nucleus and downregulation of critical tumor suppressive miRNAs 15,40. The impaired miRNA biogenesis can be restored by Pin1 inhibitor API-1, thus suppressing HCC progression 15. Since API-LP exerts better anti-HCC activity than free API-1, as shown in the above, API-LP is expected to be more powerful to regulate miRNA expression.

**Figure 4:**
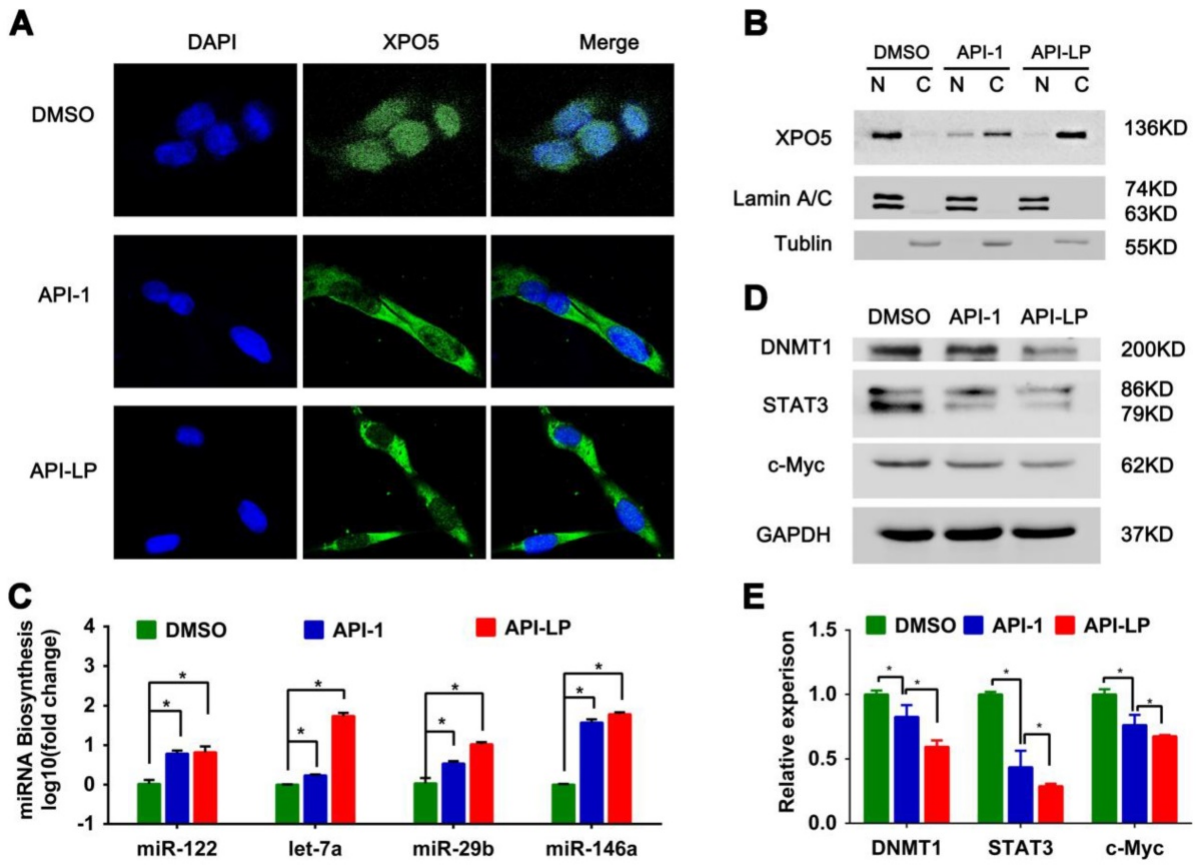
** API-LP increases the nucleus-to-cytoplasm export of XPO5 and pre-miRNAs and downregulates the expression of oncogenes.** (A-B) Confocal imaging (A) and Western blotting assay (B) of XPO5 subcellular distribution in SK-Hep1 cells treated with DMSO, API-1 or API-LP (1 μM). (C) Relative expression of mature miRNAs, detected by quantitative RT-PCR, in SK-Hep1 cells incubated with DMSO, API-1 or API-LP (1 μM). Data were shown as the means ± SD, n=3. *p < 0.05. (D-E) Immunoblotting (D) and quantitative analysis (E) of DNMT1, STAT3, c-Myc, and GAPDH expression in SK-Hep1 cells treated with DMSO, API-1 or API-LP (1 μM).

To address this question, we firstly investigated the influence of API-LP on the nucleus-cytoplasm distribution of XPO5 by performing confocal microscopy of SK-Hep1 cells treated with DMSO, free API-1 and API-LP. As expected, both API-1 and API-LP restored the export of XPO5 to cytoplasm (Figure 4A). Moreover, the cellular fractionation followed by Western blotting indicated that API-LP exhibited stronger ability than free API-1 to restore the nucleus-to-cytoplasm export of XPO5 (Figure 4B), but had little influence on XPO5 expression (Figure S2).

Considering that XPO5 is an indispensable nucleus-to-cytoplasm transport vehicle for pre- miRNAs, we subsequently investigated whether API-LP could affect the biogenesis of mature miRNAs. Based on the abundance and important biological activity during HCC development, we chose the representative miRNAs, such as miR-122, let-7a, miR-29b and miR-146a, to characterize miRNA biogenesis. MiR-122 is the most abundant miRNA in the adult liver and plays a central role in liver biology and disease. It's well documented that miR-122 acts as a tumor suppressor in the liver through targeting a variety of genes, such as c-Myc, cyclin G1, IGF1R, and Wnt1 41,42. Let-7 family inhibits the proliferation of HCC cells by downregulating the oncogenic transcription factor c-Myc and upregulating cell-cycle inhibitor p16 (INK4A) 43-45. Additionally, miR-29b plays a suppressive role in cancer cells by targeting STAT3 expression 46 and also induces global DNA hypomethylation by regulating DNMT1 levels, thus activating expression of tumor suppressor genes 47. The quantitative real-time PCR data showed that API-LP treatment significantly upregulated the expression of these miRNAs in SK-Hep1 cells when compared to free API-1 (Figure 4C).

Because miRNAs negatively regulate the expression of their target genes, the increased miRNA expression by API-LP resulted in the decreased expression of their targets, such as DNMT1, STAT3 and c-Myc (Figure 4D-E), further supporting that API- LP has the advantages over free API-1 to inhibit HCC cell growth through repairing miRNA biogenesis.

### The pharmacokinetics of API-LP in mice

**Figure 5:**
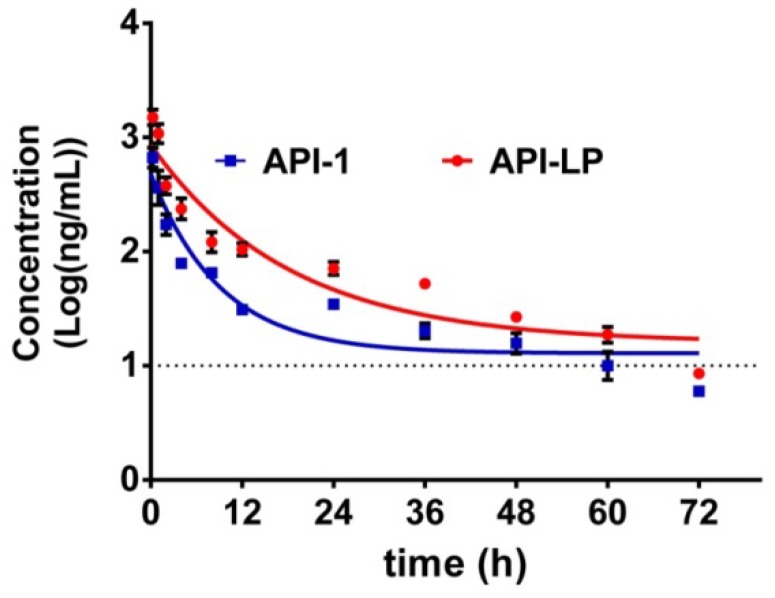
** Concentration-time profiles of API-1 following administration of 10 mg/kg API-LP or free API-1 in BALB/c mice.** Data were presented as mean ± SD, n=3.

The pharmacokinetics of API-1 were assessed following an intravenous (*i.v.*) administration of API-LP and free API-1, respectively, at a dose of 10 mg/kg in mice. The concentration-time profiles of API-1 were provided in Figure 5. Pharmacokinetic parameters including AUC, half-life (t_1/2_) and clearance were presented in Table 2. The API-1 plasma concentration following free API-1 administration decreased rapidly to 34.8 ng/mL at 24 h postdose, while the concentration following API-LP administration dropped slower to 71.81 ng/mL at 24 h postdose and was higher at all other sampling time points than free API-1. Entrapping of API-1 in the liposome greatly extended the drug residence (half-life 12.65 versus 6.31 h) and increased the systemic drug exposure (AUC_0-72_ 6.24 versus 2.61 mg/mL × h). Adding liposome encapsulation slowed down the clearance of API-1 from 72.75 ml/h to 32.06 mL/h. These pharmacokinetic results indicated an improved bioavailability of API-LP compared to free API-1.

### Safety evaluation of API-LP

**Figure 6:**
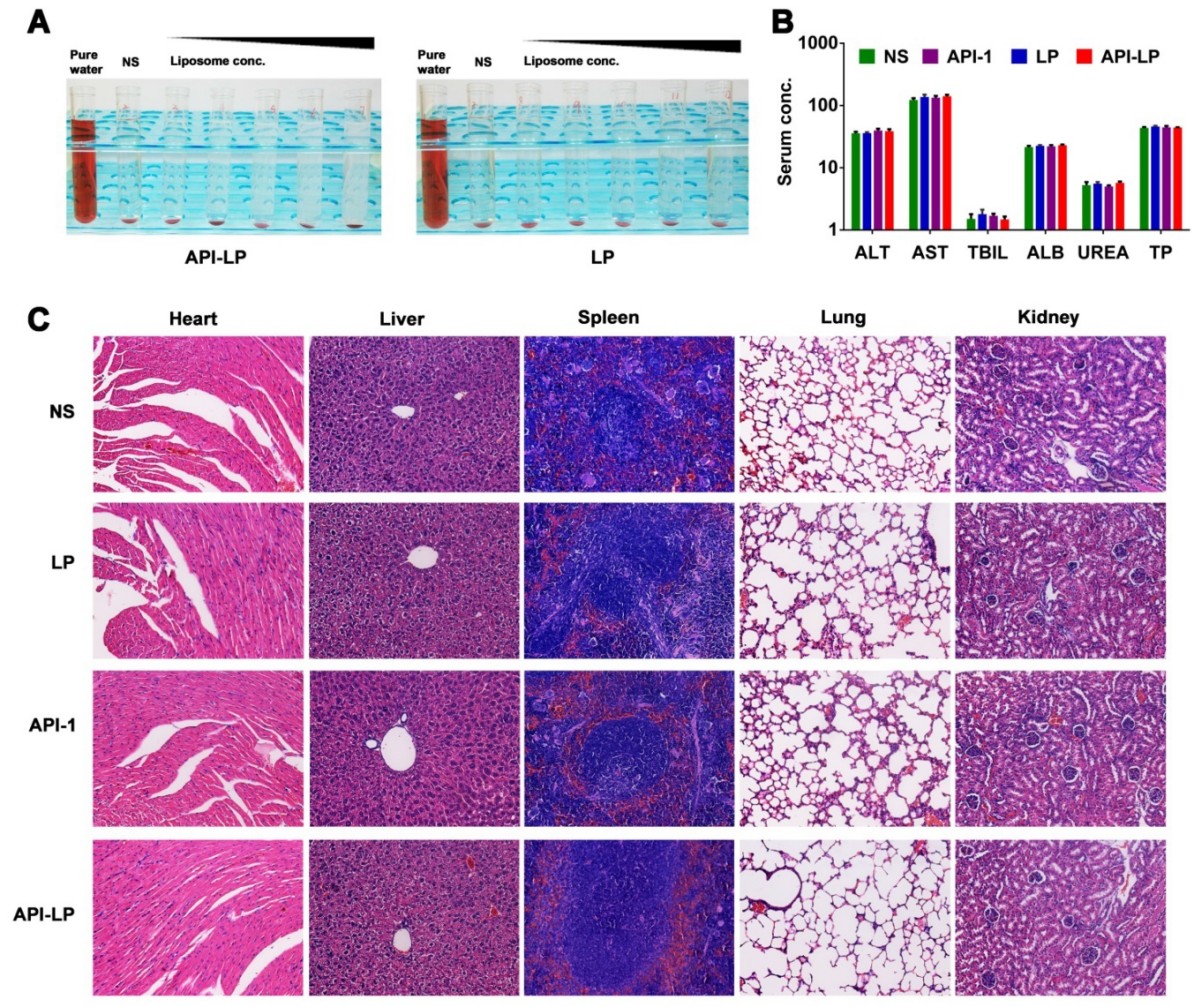
** Safety evaluation of API-LP.** (A) Photograph of hemolysis of API-LP (left) and LP (right) in various lipid concentrations. (B) Blood biochemical parameters, including ALT, AST, TBIL, ALB, UREA and TP, in mice treated with normal saline (NS), LP, API-1 or API-LP at a dose of 100 mg/kg for API-1 or API-LP. (C) H&E staining of main organs in BALB/c mice after the treatment with normal saline (NS), LP, API-1 or API-LP at a dose of 100 mg/kg for API-1 or API-LP.

Both* in vitro* hemolysis assay and *in vivo* pathological study were performed to evaluate API-LP safety. The <5% hemolysis, determined by hemoglobin concentration in rabbit blood specimen, indicated a good hemocompatibility of both API-LP and LP liposomes (Figure 6A). For *in vivo* safety evaluation, the female BALB/c nude mice were administrated via *i.v.* injection with normal saline (NS), LP, free API-1 or API-LP. Because 5 mg/kg is the efficacious dose of free API-1 to suppress HCC tumor growth in mice 15, the high dose of 100 mg/kg was selected for assessment of API-LP toxicity. The blood biochemistry data in mice showed that neither free API-1 nor API-LP had haematological or liver toxicity (Figure 6B). The H&E staining revealed that there were no apparent necrotic cells or tissues in the major organs, including heart, liver, spleen, lung and kidney (Figure 6C). Moreover, the adverse effects such as vomiting and diarrhea and significant change in mouse body weight were not observed (data not shown). Thus, API-LP formulation at single 100 mg/kg dose is safe.

With confirmation of safety and considering the favorable pharmacokinetic performance, API-LP at 10 mg/kg or lower dose level can be used for the following treatment in xenograft mouse model bearing human HCC tumors.

### Enhanced anti-HCC activity by API-LP in xenograft mouse model

**Figure 7:**
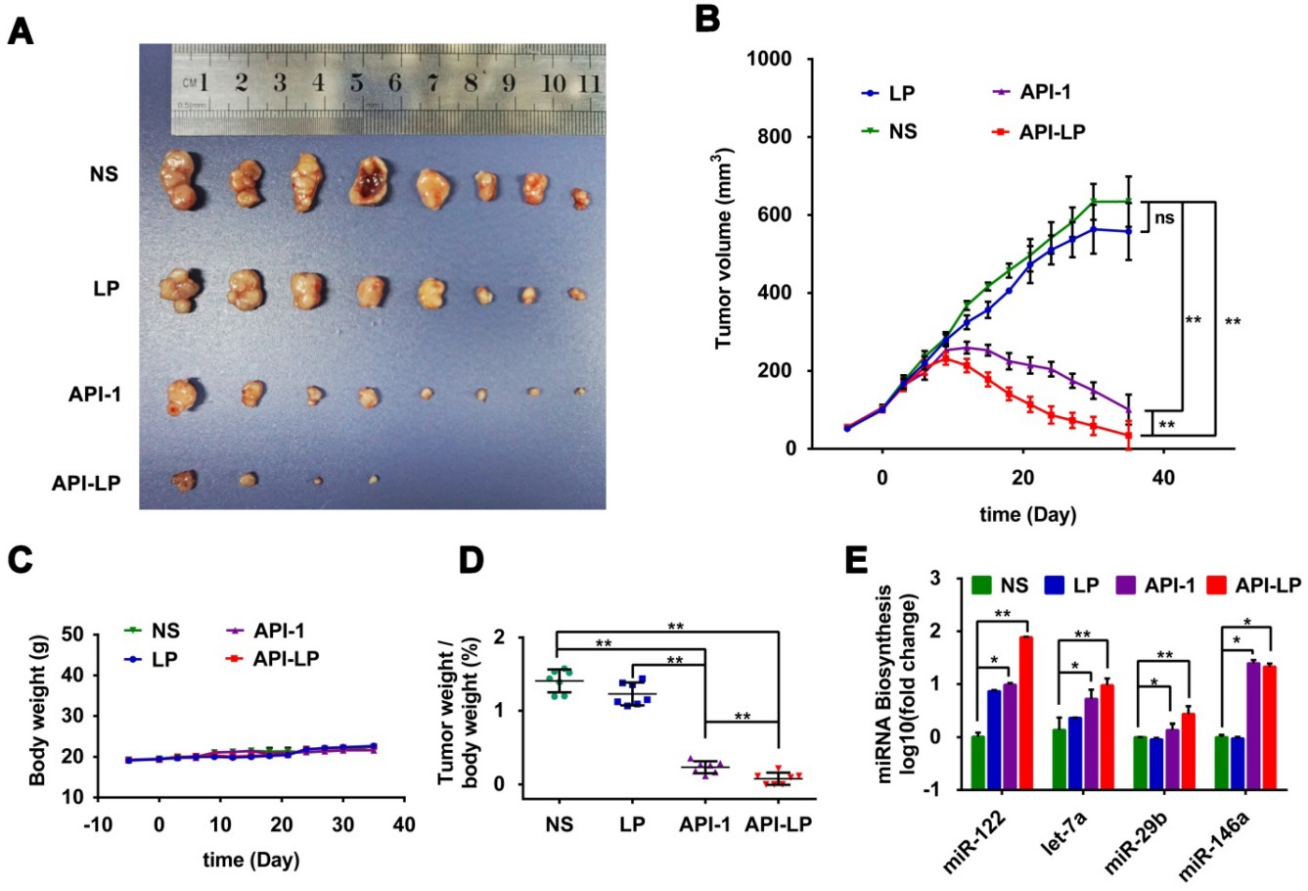
** Enhanced anti-HCC activity by API-LP in xenograft mice model.** (A) Images for SK-Hep1 tumors in BALB/c nude mice at the end of treatment with normal saline (NS), LP, API-1 or API-LP at a dose of 4 mg/kg for API-1 and API-LP. (B) SK-Hep1 tumor volumes in BALB/c nude mice at different time points. (C-D) Body weight (C) and tumor weight/body weight ratios (D) of SK-Hep1 tumor-bearing BALB/c nude mice treated with normal saline (NS), LP, API-1 or API-LP at a dose of 4 mg/kg for API-1 and API-LP. (E) Relative expression of mature miRNA detected by real-time quantitative PCR in the SK-Hep1 tumor tissues of nude mice treated with normal saline (NS), LP, API-1 or API-LP at a dose of 4 mg/kg for API-1 and API-LP. Graphic data were run in triplicate and shown as the mean ± SD. ns p ≥ 0.05, * p < 0.05, ** p < 0.01.

The *in vitro* studies above demonstrated the enhanced effect of API-LP, compared to free API-1, against HCC cells with high Pin1 expression and high XPO5 phosphorylation. To further determine the *in vivo* efficacy of API-LP, we established xenograft models with human SK-Hep1 cells inoculated in nude mice. The model mice were treated with normal saline, LP control, free API-1 control, or API-LP (N=8 for each treatment group) once every two days at 4 mg/kg dose of API-1, a lower dose than 5 mg/kg that has been identified to be effective for free API-1 to suppress SK-Hep1 tumor in the implanted mice 15. As shown in Figures 7A to 7D, both API-LP and free API-1 significantly suppressed tumor growth and reduced tumor volume but did not impact body weight. At the end of treatment (Day 35 after treatment), four mice in API-LP group had complete response with tumors gone (Figure 7A). The tumors in free API-1 group were also significantly suppressed but with less effect than those in API-LP group. The tumors in normal saline and LP control groups grew exponentially over the treatment duration (Figures 7A, 7B and 7D). Mouse body weight was not impacted by API-LP and API-1 treatment (Figure 7C), further verifying that API-LP showed no apparent toxicity and adverse effect for multiple doses and long term treatment.

Consistent with what has been observed *in vitro*, the anti-HCC effect of API-LP or free API-1 in xenograft mice was accompanied by the significantly increased biogenesis of miR-122, let-7a, miR-29b and miR-146a (Figure 7E).

**Figure 8:**
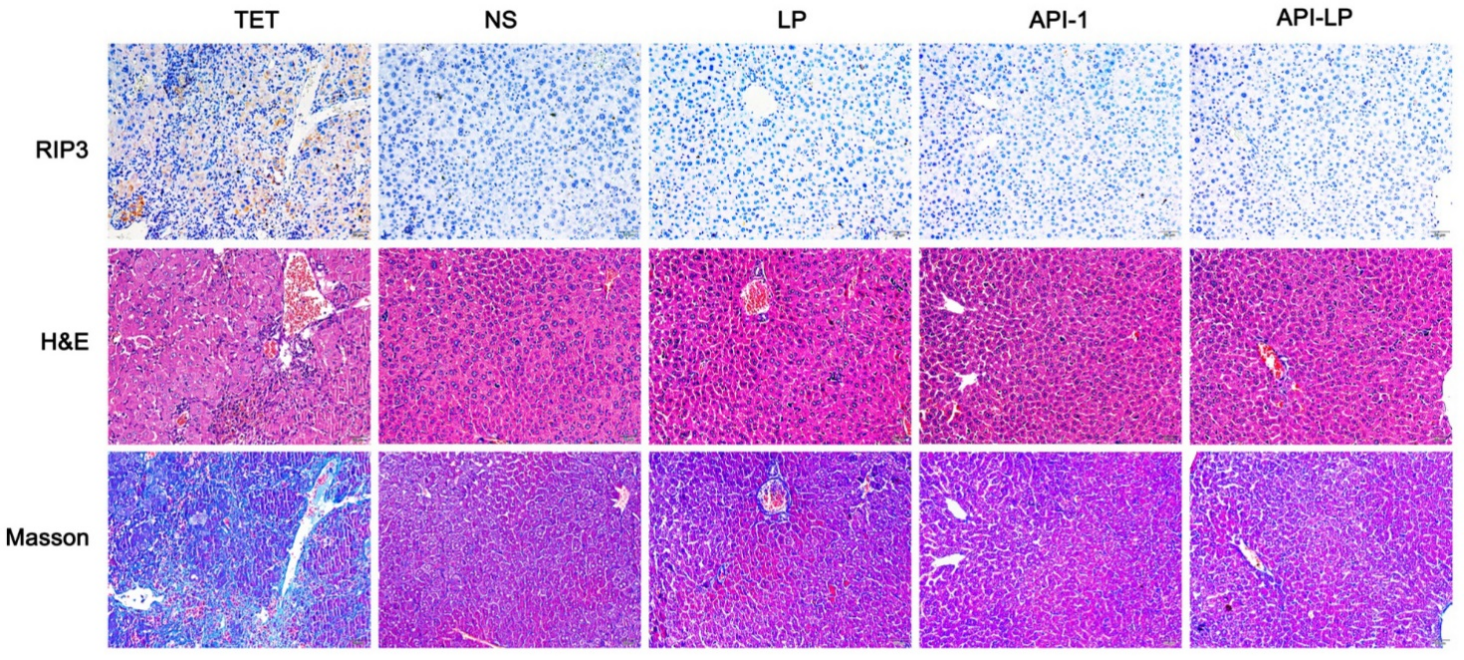
** Hepatic histopathological examinations of liver tissue from mice treated by API-LP and control formulations.** Representative images of hepatic histopathological examinations including immunostaining with anti-RIP3 antibody, H&E staining and Masson staining of mouse liver tissues after continuous administration with tetracycline (TET), normal saline (NS), LP, API-1 or API-LP at a dose of 4 mg/kg for API-1 and API-LP in tumor-bearing mice.

Moreover, the measurement of blood biochemical indicators showed no significant difference in ALT, AST and TBIL among all groups, suggesting no obvious toxicity of API-LP toward mice blood circulation and liver (Table 3). The limited liver toxicity was verified by hepatic histopathological examination (Figure 8). The H&E staining and RIP3, a cell necrosis marker, immunostaining revealed that there were no apparent necrotic cells or tissues, and the Masson staining showed no significant hepatitis and liver fibrosis in the samples (Figure 8). These toxicity assessment results following multiple dosing of 4 mg/kg API-LP in tumor-bearing mice were consistent with the results following a single 100 mg/kg dose in BALB/c nude mice and further verified the safety of API-LP. Taken together, API-LP exerts good anti-HCC activity *in vivo* without apparent toxicity.

## Conclusions

In this study, a novel liposomal formulation of Pin1 inhibitor API-1, named as API-LP, was developed for the purpose of enhancing the anti-cancer activity through an efficient delivery of API-1 to HCC cells. It exhibited small particle size (241.5 nm) with approximately 65% encapsulation efficiency of API-1 and excellent stability over storage. Moreover, compared to free API-1, API-LP showed favorable biocompatibility, increased exposure in mouse blood circulation, and enhanced anti-HCC activity *in vitro* and in xenograft mice model. Mechanistically, API-LP exerted better activity than free API-1 to inhibit Pin1, thus enhancing nucleus-to-cytoplasm export of XPO5, promoting miRNA expression to downregulate expression of miRNA-targeting oncogenes, such as DNMT1, STAT3 and c-Myc. The above findings suggested a novel liposomal system of API-1 as a promising therapeutic strategy for HCC treatment.

## Supplementary Material

Supplementary figures.

## Figures and Tables

**Table 1:** The characteristics of liposomal formulations with varied lipid compositions

Liposomal Formulation	Lipids Composition (M)	API-1/Lipids (w/w)	Mean±SD Diameter (nm)	Mean±SD PDI	Mean±SD Zeta Potential (mV)	EE (%)
API-LP1	DMPC/DMPG/CHOL/DSPE-PEG_2000_ 25:25:45:5	20%	233.8±1.65	0.214±0.018	-12.6±0.18	37.7
API-LP2	DMPC/DMPG/CHOL/DSPE-PEG_2000_ 22.5:22.5:50:5	20%	229.3±0.43	0.227±0.006	-12.7±0.58	56.9
API-LP3	DMPC/DMPG/CHOL/DSPE-PEG_2000_ 20:20:55:5	20%	232.4±0.42	0.281±0.046	-12.4±0.34	46.9
API-LP4	DMPC/DMPG/CHOL/DSPE-PEG_2000_ 22.5:22.5:50:5	30%	241.5±1.33	0.273±0.007	-11.5±0.25	65.2
API-LP5	DMPC/DMPG/CHOL/DSPE-PEG_2000_ 22.5:22.5:50:5	40%	268.1±1.74	0.309±0.032	-10.0±0.66	42.9
LP	DMPC/DMPG/CHOL/DSPE-PEG_2000_ 22.5:22.5:50:5	0%	86.6±0.20	0.195±0.020	-13.4±0.07	—

EE: encapsulation efficiency.

**Table 2:** Pharmacokinetic parameters

Pharmacokinetic Parameters	API-LP Estimates	API-1 Estimates
AUC_0-24_ (mg/mL × h)	4.58 ± 0.11	1.80 ±0.07
AUC_0-48_ (mg/mL × h)	5.80 ± 0.11	2.35 ±0.10
AUC_0-72_ (mg/mL × h)	6.24 ± 0.13	2.61 ±0.13
Half-life t_1/2_ (h)	12.65 ± 2.93	6.31 ±0.66
Clearance (mL/h)	32.06 ± 0.68	72.75 ±3.93

AUC: concentration-time curve.

**Table 3:** Blood biochemistry

	ALT (U/L)			AST (U/L)			TBIL (μmol/L)		
	Mean	SD	*p*-value	Mean	SD	*p*-value	Mean	SD	*p*-value
**NS**	35.83	5.34	-	122.50	20.93	-	1.51	0.72	-
**LP**	36.00	3.16	0.95, ns	137.50	29.81	0.34, ns	1.80	0.78	0.52, ns
**API-1**	39.67	6.77	0.30, ns	133.67	22.47	0.39, ns	1.70	0.32	0.57, ns
**API-LP**	38.67	7.50	0.47, ns	141.00	19.99	0.15, ns	1.48	0.43	0.93, ns

ALT: alanine transaminase; AST: aspartate transaminase; TBIL: total bilirubin.

## Abbreviations

HCC: hepatocellular carcinoma
PDI: polydispersity
EE: encapsulation efficiency
miRNA: microRNA
pre-miRNA: precursor miRNA
XPO5: exportin-5
EGCG: epigallocatechin gallate
ATRA: all trans retinoic acid
API-LP: liposome-encapsulated API-1
LP: empty liposomal formulation
DMPC: 1,2-Dimyristoly-sn-glycero-3-phosphocholine
DMPG: 1,2-dimyristoyl-sn-glycero-3-[phospho-rac-(1-glycerol)]
DSPE-PEG_2000_: 1,2-distearoyl-sn-glycero-3-phosphoethanolamine-N-[amino(polyethylene glycol)-2000]
CHOL: cholesterol
EE: encapsulation efficiency
DLS: dynamic light scattering
TEM: transmission electron microscope
HPLC: high performance liquid chromatography
DSC: differential scanning calorimetry
MTT: 3-(4,5-dimethylthiazol-2-yl)-2,5-diphenyltetrazolium bromide
H&E: hematoxylin and eosin
PI: propidium iodide
PFA: phosphate buffered paraformaldehyde
ALT: alanine transaminase
AST: aspartate transaminase
TBIL: total bilirubin
ALB: albumin
UREA: urea nitrogen
TP: total protein
DAPI: 4,6-diamidino-2-phenylindole
AUC: concentration-time curve
*i.v.*: intravenous
NS: normal saline
