# Supplementary material for: MicroRNA Biogenesis is Enhanced by Liposome-Encapsulated Pin1 Inhibitor in Hepatocellular Carcinoma
Source: Theranostics. 2019 Jul 9;9(16):4704–16. doi: 10.7150/thno.34588 (PMC6643437; doi:10.7150/thno.34588)
Supplement: Supplementary file 1 — Supplementary figures. [file thnov09p4704s1.pdf]

MicroRNA Biogenesis is Enhanced by Liposome-encapsulated Pin1 Inhibitor in Hepatocellular Carcinoma

Dan Sun,[1,#] Shuangyan Tan,[1,#] Yanli Xiong,[1,2] Wenchen Pu,[1] Jiao Li,[1] Wei Wei,[2] Canhua Huang,[1,3] Yu-Quan Wei,[1] Yong Peng[1,*]

[1] *State Key Laboratory of Biotherapy and Cancer Center, National Clinical Research Center for Geriatrics, West China Hospital, Sichuan University, Chengdu, 610041, Sichuan, China.*

[2] *Key Laboratory of Bio-Resource and Eco-Environment of Ministry of Education, College of Life Sciences, Sichuan University, Chengdu 610065, China.*

[3] *West China School of Basic Medical Sciences & Forensic Medicine, Sichuan University, Chengdu 610041, China.*

Supplementary Information

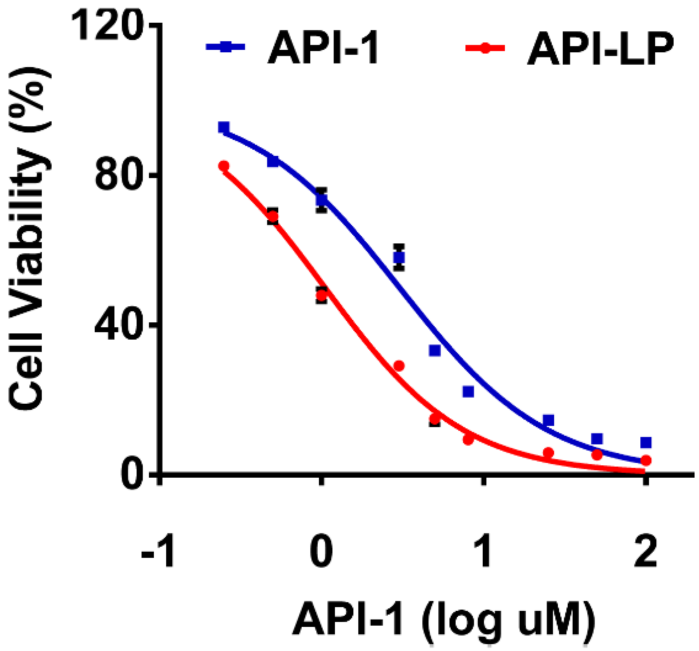

Figure S1. MTT assays after API-1 (blue) and API-LP (red) treatment of SK-Hep1 cells. Data are presented as mean ± SD, n=3.

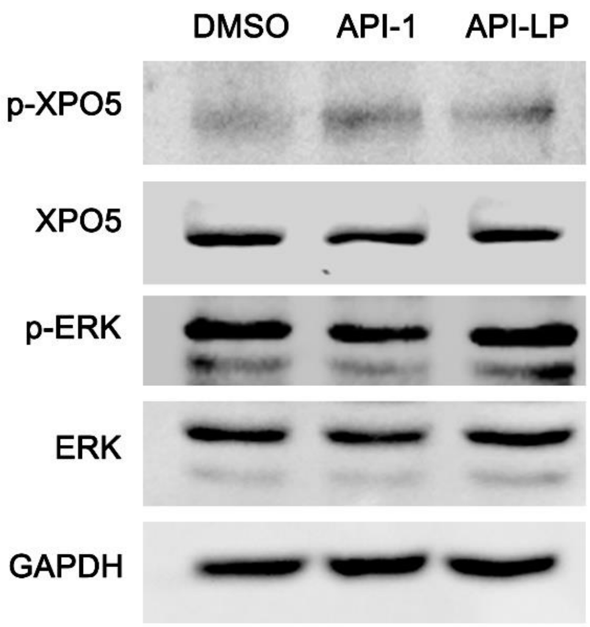

Figure S2. Immunoblotting analyses of XPO5, p-XPO5, ERK, p-ERK, and GAPDH levels in SK-Hep1 cells treated with DMSO, API-1 or API-LP (1 μM).
